# Prenatal Protein Malnutrition Produces Resistance to Distraction Similar to Noradrenergic Deafferentation of the Prelimbic Cortex in a Sustained Attention Task

**DOI:** 10.3389/fnins.2019.00123

**Published:** 2019-02-19

**Authors:** Lori A. Newman, Jaime Baraiolo, David J. Mokler, Arielle G. Rabinowitz, Janina R. Galler, Jill A. McGaughy

**Affiliations:** ^1^Department of Psychology, University of New Hampshire, Durham, NH, United States; ^2^Department of Psychological Science, Vassar College, Poughkeepsie, NY, United States; ^3^Department of Biomedical Sciences, College of Osteopathic Medicine, University of New England, Biddeford, ME, United States; ^4^Department of Neurology and Neurosurgery, McGill University, Montreal, QC, Canada; ^5^Department of Psychiatry, Harvard Medical School, Boston, MA, United States; ^6^Division of Pediatric Gastroenterology and Nutrition, Mucosal Immunology and Biology Research Center, MassGeneral Hospital for Children, Boston, MA, United States

**Keywords:** cognition, prefrontal cortex, distractibility, norepinephrine, rat model of malnutrition

## Abstract

Exposure to malnutrition early in development increases likelihood of neuropsychiatric disorders, affective processing disorders, and attentional problems later in life. Many of these impairments are hypothesized to arise from impaired development of the prefrontal cortex. The current experiments examine the impact of prenatal malnutrition on the noradrenergic and cholinergic axons in the prefrontal cortex to determine if these changes contribute to the attentional deficits seen in prenatal protein malnourished rats (6% casein vs. 25% casein). Because prenatally malnourished animals had significant decreases in noradrenergic fibers in the prelimbic cortex with spared innervation in the anterior cingulate cortex and showed no changes in acetylcholine innervation of the prefrontal cortex, we compared deficits produced by malnutrition to those produced in adult rats by noradrenergic lesions of the prelimbic cortex. All animals were able to perform the baseline sustained attention task accurately. However, with the addition of visual distractors to the sustained attention task, animals that were prenatally malnourished and those that were noradrenergically lesioned showed cognitive rigidity, i.e., were less distractible than control animals. All groups showed similar changes in behavior when exposed to withholding reinforcement, suggesting specific attentional impairments rather than global difficulties in understanding response rules, bottom-up perceptual problems, or cognitive impairments secondary to dysfunction in sensitivity to reinforcement contingencies. These data suggest that prenatal protein malnutrition leads to deficits in noradrenergic innervation of the prelimbic cortex associated with cognitive rigidity.

## Introduction

Malnutrition impacts approximately one in four children worldwide (WHO, 2012). Longitudinal studies of adults with exposure to prenatal food restriction during the Dutch famine and the Chinese famine of 1959–1961 (St. Clair et al., 2005) have shown an increased prevalence of neuropsychiatric disorders (Brown et al., 1995; Brown and Susser, 2008) and attentional impairments (De Rooij et al., 2010; Li et al., 2015). Similarly, a longitudinal study of Barbadian adults exposed to protein-calorie malnutrition limited to the first year of life has documented impaired attention (Galler et al., 2012), and affective processing (Waber et al., 2014). Attentional problems were evident in this cohort across the life span, as confirmed by parent, and teacher ratings (Galler et al., 1983, 1990; Galler and Ramsey, 1989) as well as self-reports in middle adulthood (Galler et al., 2012). Neuropsychological testing showed that deficits in attention, as assessed using the Behavioral Rating Inventory of Executive Function (BRIEF), the Wisconsin Card Sorting Task (WCST; Waber et al., 2014), and a continuous performance task (Galler et al., 2012), persisted well into middle adulthood. This study also confirmed that exposure to early childhood malnutrition entailed long-lasting epigenetic signatures in the Barbados cohort that were closely associated with the attention problems, even after adjusting for socioeconomic and ecologic conditions in the household (Peter et al., 2016). However, consistent and compelling data from human studies are often complicated by a multitude of other factors that coincide with childhood malnutrition, e.g., poverty, infection, stress, and maternal depression (Salt et al., 1988; Galler et al., 2000; Walker et al., 2011). While these long-term effects are hypothesized to result from dysregulation of the prefrontal cortex, it is difficult to ascertain the relationship between prenatal malnutrition and the prefrontal cortex in human studies. Animal models that reproduce these conditions are therefore better suited to elucidate causal relationships among malnutrition, cognition, and changes in the brain (Tonkiss et al., 1993; Galler et al., 1996). Animal models have shown impaired attentional processing as a result of prenatal iron deficiency (Mohamed et al., 2011), vitamin D (Turner et al., 2013), and protein levels (McGaughy et al., 2014). Moreover, these nutritional deficits are also known to impair prefrontal circuits hypothesized to be critical to attention (Groves et al., 2013; Grissom et al., 2014; McGaughy et al., 2014).

The present studies investigated the impact of prenatal malnutrition on sustained attention and distractibility using a previously validated task (McGaughy and Sarter, 1995; Demeter et al., 2008; Newman et al., 2008). Prior studies using a well-defined animal model of prenatal protein malnutrition revealed cognitive rigidity in a test of attentional set shifting (McGaughy et al., 2014). We hypothesized another outcome of this rigidity would be that prenatally malnourished rats would be less sensitive to the detrimental effects of a distractor than controls. Cognitive rigidity can result from decreased functioning of prefrontal noradrenergic systems (Tait et al., 2007; Newman et al., 2008; McGaughy et al., 2014), while increased distractibility may result from hypofunctioning of the cholinergic systems (Newman et al., 2008; Berry et al., 2014). Because of the critical role of these two neuromodulatory systems in attentional processing, we performed histological analyses on these systems upon completion of behavioral testing. Rats exposed to prenatal malnutrition had fewer noradrenergic afferents in the prelimbic cortex relative to control subjects, but cholinergic afferents were unchanged by prenatal malnutrition. Based on this finding, we assessed the effects of selective noradrenergic deafferentation of the prelimbic cortex in adult rats on distractibility to compare with the effects of prenatal protein malnutrition. We hypothesized that noradrenergic deafferentation would reproduce cognitive rigidity found in subjects exposed to prenatal malnutrition.

## Materials and Methods

### Prenatal Nutritional Treatment

For the prenatal malnutrition studies, viral-free virgin, female, Long-Evans hooded rats (175–200 g) (obtained from Charles River, Wilmington, MA, United States) were randomly assigned to one of two nutritional conditions. As described in detail previously (Galler and Tonkiss, 1991), one group was placed on an adequate protein diet, 25% casein (Teklad Laboratories, Madison, WI, United States); 5 weeks prior to mating and throughout pregnancy (Fischer et al., 2015), while the other group received an isocaloric, low protein diet, 6% casein (Teklad Laboratories, Madison, WI, United States) during the same period. Beginning the experimental diets prior to pregnancy ensured that there was no impact on food intake during pregnancy as a result of the diet change and was more representative of human malnutrition which is most often a chronic state (Galler and Tonkiss, 1991). All females were mated with males that had been acclimated to these respective diets for 1 week. Throughout pregnancy, dams were singly housed in individual polysulfone breeding cages (39.5 × 34.6 × 21.3 cm; Tecniplast USA Inc., Exton, PA, United States). Following parturition, litters from both nutritional groups were culled to eight pups (two females and six males) and fostered as whole litters to well-nourished lactating foster dams receiving the 25% casein diet (Table 1). Each foster dam had given birth within the same 24 h period. At birth, pups born to mothers on the 6% casein diet that were fostered to mothers on the 25% casein diet were designated as members of the 6/25 (prenatally malnourished) group, while pups born to mothers on a 25% casein diet that were also fostered to mothers on a 25% casein diet were designated as members of the 25/25 (prenatally well-nourished or control) group. All dams and litters were provided with *ad libitum* access to the 25% casein diet during the litter period. At postnatal day (PND) 21, all rats were weaned, placed on a standard laboratory chow diet containing 23% protein (Purina Mills Inc., Richmond, IN, United States; Formula 5001) and pair housed with littermates in polysulfone breeding cages (Tecniplast USA Inc., Exton, PA, United States). One week prior to behavioral assessment, subjects were single-housed and began food restriction.

**Table 1:** Schematic of prenatal nutritional treatment groups.

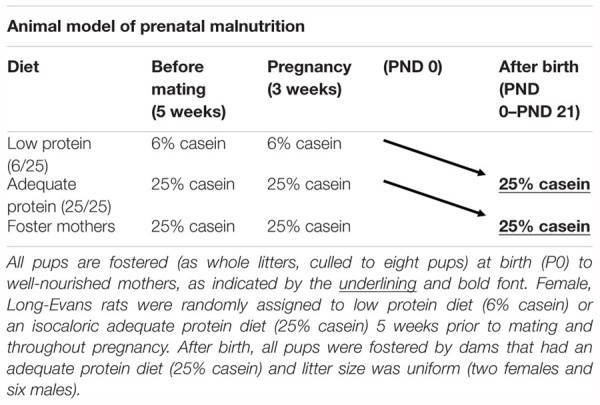

### Subjects

For the prenatal nutrition animals, the vivarium was maintained at 22–24°C with 40–60% humidity and kept on a 12:12 h reverse light/dark cycle with lights on at 19:00 h to accommodate to the waking state of the rats. During the dark cycle, red florescent lighting provided continuous dim illumination. Behavioral testing started at PND 90 and occurred during the dark phase of the cycle between the hours of 9:00 and 13:00 h, 6 days per week. One male rat from each of 10 6/25 prenatally malnourished litters and 17 25/25 control litters served as subjects and were singly housed in polycarbonate cages. In no instance were littermates tested. The norepinephrine (NE) lesion study used 24 adult male Long Evans rats (Harlan, Indianapolis, IN, United States) housed separately, kept on a 12:12 h light/dark cycle (lights on at 6 am) in a climate-controlled environment, and only tested during the light hours.

All subjects received ∼18 g of standard rat chow daily to allow them to maintain weights that were approximately >90% of age-matched controls. Water was available *ad libitum.* All animals were weighed weekly to assure healthy weights relative to age-matched controls. All personnel involved in collecting behavioral and weight data were blind to condition during data collection. Procedures were approved by the University of New Hampshire Institutional Animal Care and Use Committee and the University of New England Institutional Animal Care and Use Committee in accordance with guidelines outlined in *Guide for the Care and Use of Laboratory Animal (Approval No. 20101005MOK)*.

### Apparatus and Materials

Operant chambers (Med Associates, St. Albans, VT, United States) equipped with two retractable levers, a houselight (2.8 W), a 45 mg pellet dispenser, a 2,900-Hz sonalert tone generator, and three panel lights (2.8 W) were used. Each chamber was outfitted with two retractable response levers mounted 11 cm apart with associated stimulus light. The third panel light was centered between the two other lights above the food hopper. A houselight, located at the top of the back panel of each operant chamber, provided ambient illumination during a test session. A pellet dispenser delivered reinforcers (Bioserv, 45 mg; Research Diets, Frenchtown, NJ, United States or Noyes Precision Pellets, 45 mg; Research Diets, New Brunswick, NJ, United States) into a food hopper, located halfway between both response levers. The food hopper, panel lights, tone generator and retractable levers were all located on the same wall, whereas the houselight was located on the opposite wall. Signal presentation, lever operation, and food pellet delivery were recorded using a PC with Windows XP and the Med-PC IV software (Med Associates, St. Albans, VT, United States).

### Behavioral Training

Rats were initially trained to bar press for food in the operant chamber in accordance with an FR1 schedule of reinforcement with the houselight illuminated. Reinforcement was suspended when the rat pressed one lever over five times more than the other lever to prevent the development of a side bias. Once the animals made at least 50 responses for two consecutive days, training in the sustained attention task (SAT) was begun.

#### Sustained Attention Task (SAT): Shaping

After learning to bar press for food, the animals were trained to discriminate between signal and non-signal trials as described in previous studies (Newman et al., 2008). Training sessions consisted of a total of 162 trials. Rats were placed into the operant chambers for 1 min prior to the onset of training. The houselight remained illuminated for the duration of the session. Signal and non-signal trials were presented in a pseudo-randomized sequence so that each block of 54 trials consisted of an equal number of signal and non-signal events. Signal trials consisted of illuminating the central and left panel lights for 1 s, whereas the lights were not illuminated for non-signal trials. Animals were cued to respond by the extension of both levers into the box two seconds after the signal or non-signal event. Levers remained extended for 4 s or until a lever press occurred. Animals were reinforced for responding to the light stimuli by pressing the left lever (hit) and by pressing the right lever in the absence of the light (correct rejection). Incorrect lever presses were defined as misses when they occurred on a signal trial and false alarms when they occurred on a non-signal trial. If the animal failed to respond or responded incorrectly, the levers were retracted and the inter-trial interval (ITI; 12 ± 3 s) was reinstated. After an incorrect response, the trial was repeated up to three times (correction trials). If the animal failed to respond correctly after three correction trials, a forced-choice trial was initiated. In forced-choice trials, the event (signal or non-signal) was repeated but only the correct lever was extended and remained active for 90 s. On forced-choice, signal trials, the lights remained illuminated for 90 s. These trials facilitated discriminative conditioning and prevented the development of a side bias. After the animals responded correctly to ≥70% of both the signal and non-signal events for at least two consecutive testing days, they participated in a second shaping task. During this task, the central panel light was only illuminated for 1 s during signal trials. All other aspects of the task were the same as the previous shaping task. After the animals responded correctly to ≥70% of both the signal and non-signal events for at least two consecutive testing days in this phase of shaping, they entered the final baseline task that served as the comparator for all tests of altered attentional demand.

#### Baseline Sustained Attention Task (SAT)

In the final version of the SAT, the length of the signal duration was changed from 1 s to pseudorandom presentation of 25, 100, and 500 ms. Sessions consisted of 27 trials of each of the three signal lengths and 81 trials of the non-signal trials, yielding a total of 162 trials per session. As performance changes were analyzed across three blocks of 54 trials each, the sequence of signal and non-signal trials was pseudo-randomized so that one block consisted of 27 signal and 27 non-signal trials with each signal length being presented nine times. In addition, both correction and forced-choice trials were discontinued. Animals were trained to a criterion of >70% hits to the 500 ms signals and >70% correct rejections to non-signal trials for at least two consecutive sessions, at which point they were considered ready to undergo tests of altered attentional demand in the prenatal malnutrition study or ready for surgery (see the section “Surgery”) in the noradrenergic prelimbic lesion study. Tests of varied attentional demand were counterbalanced across subjects to control for possible practice and order effects.

#### Effects of Distracting Visual Stimuli

To allow comparison with previously published studies (McGaughy and Sarter, 1995; McGaughy et al., 1997; Newman et al., 2008), we assessed the effects of flashing the houselight in a predictable pattern for one session (0.5 Hz, Predictable Distractor, dSAT) or an unpredictable pattern with an average on/off cycle similar to the 0.5 Hz (0.25, 0.5, 1.0, 1.5, 2.0, or 3.0 s on/off; Unpredictable Distractor, uSAT). Additionally, as previous work in our laboratory has shown that lesions to the posterior parietal cortex increase susceptibility to task irrelevant stimuli that are identical in duration to those of the target stimuli (Newman and McGaughy, unpublished data), we also assessed the effects of this type of distractor in the present study (Overlapping Distractor; 0.025, 0.1, 0.5 s on/off, oSAT).

#### Effects of Withholding Reinforcement

As prenatal protein restriction has been shown to influence sensitivity to reward (Morgane et al., 1993; Tonkiss et al., 1993), we directly assessed the effect of withholding reinforcement on attentional performance in the SAT (SATwr) by omitting reinforcement after correct responses.

#### Behavioral Measures

For each test session, the number of hits, misses, correct rejections, false alarms and errors of omission were recorded. The relative number of hits (% hits = hits/hits + misses) was computed for each signal length along with the relative number of correct rejections (% CR = correct rejections/correct rejections + false alarms). In addition, we calculated the relative number of left lever presses (hits + false alarms/all responses) as a measure of side bias. This was done when the initial analyses of hits and correct rejections suggested such a bias could explain the pattern of results (e.g., hits were significantly increased while correct rejections were significantly decreased).

### Surgery

After learning the SAT and prior to the testing of task variants, rats in the noradrenergic lesions study underwent intracranial surgery. Subjects were anesthetized with an intramuscular (i.m.) injection of ketamine (85 mg/kg/ml) and xylazine (8.5 mg/kg/ml) then placed in a stereotaxic frame using atraumatic ear bars. Rats received either lesions of the noradrenergic afferents to the prefrontal cortex using a solution of 0.01 μg/μl dopamine beta-hydroxylase saporin (DBH-SAP) in a sterile phosphate buffer or sham-lesions produced by infusing sterile phosphate buffer into medial, prefrontal cortex (Newman et al., 2008). All infusions (0.5 μl/hemisphere) were made at the following coordinates: toothbar: -3.3; anteroposterior (AP): Bregma +2.8; mediolateral (ML): Bregma ±0.6; dorsoventral (DV): Skull -5.2 using a 26 gauge, 10 μl microsyringe attached to an electronic infusion pump (Micro 4^TM^ Microsyringe Pump Controller, World Precision Instruments, Sarasota, FL, United States). To prevent unwanted diffusion, the toxin or its vehicle was infused at a rate of 125 nl/min with the needle left in place for 4 min before and after infusion. Post-surgery animals were given 7 days of recovery time to allow for retrograde transport of the toxin and apoptotic cell death to occur. During recovery, rats were given *ad libitum* food and water.

#### Postoperative Training

Rats in the noradrenergic lesion study received 2 weeks of *ad libitum* food and water prior to the reinstatement of food restriction and the onset of post-operative behavioral testing. When rats performed at criterion performance (>75% hits 500; >75% correct rejections) for two consecutive days, variations of attentional demands began. After the completion of a testing session, rats were returned to training in the SAT and again required to perform at criterion levels in the SAT for 2 days prior to the next test of altered cognitive demand.

### Histology

Following the completion of behavioral testing, rats were deeply anesthetized with Euthasol (Virbac USA, Fort Worth, TX, United States), ex-sanguinated with 0.9% saline and then 4% paraformaldehyde in 0.1 M phosphate buffer. Perfused brains were then placed in 30% sucrose to provide cryoprotection. Sections (50 μm) were collected using a microtome (Leica, Buffalo Grove, IL, United States) attached to a freezing stage (Physitemp, Clifton, NJ, United States). Alternate sections were stained for DBH positive fibers, acetylcholinesterase positive fibers (AChE+) or Nissl bodies using thionin. To prevent uneven staining, all rinses and incubations were performed using an orbital shaker.

#### Dopamine β-Hydroxylase (DBH) Immunohistochemistry

Sections were initially placed into a solution of 1% hydrogen peroxide and 3% normal goat serum in phosphate-buffered saline (PBS). Without rinsing, sections were then transferred to a solution of 1:2000 mouse anti-DBH (EMD Millipore, Billerica, MA, United States) in 0.2% Triton X-100 in PBS and left overnight. Subsequent to 3 × 10 min rinses in PBS, sections were incubated in biotinylated secondary antibody (Goat anti-mouse, Santa Cruz Biotechnology, Dallas, TX, United States) for 2 h. After rinsing 3 × 10 min in PBS, sections were incubated in the avidin biotin complex solution (ABC; Vector Labs, Burlingame, CA, United States) for 1.5 h. Subsequent to rinsing with PBS (3 × 10 min), visualization was accomplished with a solution of nickel enhanced 3,3-diaminobenzidine (Vector Labs, Burlingame, CA, United States) until cortical layers became visible (1–5 min). Finally, sections were rinsed with PBS (3 × 10 min) prior to mounting on gelatin coated slides. Sections were dried overnight in a 37°C oven prior to dehydration, defatting, and cover-slipping.

#### Acetylcholinesterase Staining

The staining procedure used for acetylcholinesterase was modified from a protocol described previously by Tago et al. (1986). Sections were placed in phosphate buffer (pH 7.4) with 0.1% hydrogen peroxide for 30 min and then were washed in 0.1M maleate buffer (three rinses, 3 min each) in order to modify the pH to 6.0. An incubation solution of 5 mg of acetylthiocholine iodide, 0.174 g of sodium citrate, 0.075 g of copper sulfate, and 0.0164 g of potassium ferricyanide in 0.1 maleate buffer was then used to soak sections for 60 min. Sections were next washed for a total of three rinses in 50 mM Tris buffer (pH 7.6) for 3 min each. They were then soaked for 10 min in a second incubation solution of 0.05 g of diaminobenzidine and 0.375 g of nickel ammonium sulfate in 125 ml of 50 mM Tris buffer. Twelve drops of hydrogen peroxide were then added to the solution until the details of the sections became apparent. Finally, the sections were thoroughly rinsed three times in 5 mM Tris buffer (3 min each) and mounted on gelatin-coated slides after which they were dried overnight in a 37°C oven prior to dehydrating, defatting, and cover-slipping.

#### Microscopic Analyses

Brain sections were analyzed using an Olympus Bx51 microscope (Optical Analysis Corporation, Nashua, NH, United States) at 400× magnification in conjunction with a Nikon DXM 1200 camera at 10× magnification. Image Pro Plus software v.6.0 (Media Cybernetics, Silver Springs, MD, United States) was used to superimpose a grid over the brain images. The number of fibers that definitively crossed the perimeter of the grid were counted and recorded. Counts were taken in the prelimbic cortex (PL) at Bregma +4.7, +3.7, and +2.7 mm; and anterior cingulate cortex (ACC) at Bregma +3.7, +2.5, and +0.7 mm anterior.

### Statistical Analyses

All statistical analyses were performed using SPSS 25.0 (IBM, Armonk, NY, United States). The degrees of freedom in all analyses were corrected using the Huynh-Feldt correction in the case of a violation of sphericity. Epsilon (𝜀) values not equal to 1 are reported below. The prenatal nutrition study and the NE lesion study had separate controls (25/25 casein and SHAM-LX) and therefore analyses from each study were run separately.

#### Histological Analyses

Histological data were analyzed using a mixed factor ANOVA for each cortical subregion with Nutrition (two levels) or Lesion (two levels) as the between-subjects factor and the within-subjects factor of Rostral to Caudal (three levels in counts from PL; three levels in counts from ACC). Poor fixation on one subject in the prenatal malnutrition study precluded histological processing, resulting in final n’s of 9 and 17 for the 6/25 and 25/25 groups, respectively. Staining from one NE-LX rat was lost to an error in tissue processing.

#### Baseline Sessions

All dependent measures were analyzed using separate mixed factor ANOVAs. In order to determine if there was any difference based on prenatal nutritional treatment or lesioning, on performance on the SAT, baseline days prior to each test of attentional variation was compared to the other days. For the analysis of the effects of time on task (vigilance decrement), test sessions were divided into three blocks of 54 trials each (see above). The effects of signal length and block over the days of baseline on hit accuracy were analyzed using a mixed factor ANOVA with one between-subjects factor [e.g., Nutrition (2)] and three within subject factors [Day (4), Block (3), and Signal (3)]. The effects of block on correct rejection accuracy were analyzed using a mixed factors ANOVA with one between-subjects factor [e.g., Lesion (2)] and two within-subject factors [Day (4) and Block (3)].

#### Sessions With Varied Attentional Demand

Baseline performance for each dependent measure was calculated using session in the standard task (ITI: 12 ± 3 s) immediately prior to the test of altered attentional demand (e.g., 0.5 Hz Distractor), so that each ANOVA had two levels that allowed a comparison of performance in the standard SAT to the test session. The effects of varied attentional demand were assessed in independent, mixed factors ANOVAs with one between-subjects factor [Nutrition (2)] and three within subject factors [Task Variation (2), Block (3), and Signal (3)]. The effects on correct rejection accuracy were analyzed using a mixed-factors ANOVA with one between-subjects factor [Nutrition (2)] and two within-subject factors [Task Variation (2) and Block (3)]. A summary of the effects of prenatal malnutrition or noradrenergic lesions on % hits and % correct rejections can be found in Table 2.

**Table 2:** A summary of the main effects of prenatal protein malnutrition and noradrenergic lesions on the primary dependent measures from the SAT.

	Prenatal malnutrition				Noradrenergic lesion			
	% Hits		% CR		% Hits		% CR	
Test	25/25	6/25	25/25	6/25	SHAM-LX	NE-LX	SHAM-LX	NE-LX
dSAT	42.9 ± 4.2	47.4 ± 5.5	82.1 ± 1.6	85.4 ± 2.2	35.9 ± 3.6	55.9 + 3.5	83.9 + 1.8	74.8 + 2.8
uSAT	45.5 ± 3.0	57.1 ± 4.1	71.9 ± 2.6	68.0 ± 3.5	53.2 ± 4.9	54.6 ± 3.2	67.8 + 3.0	67.8 + 1.9
oSAT	45.9 ± 2.7	45.8 ± 3.5	82.1 ± 1.6	78.9 ± 2.2	n.a.	n.a.	n.a.	n.a.
SATwr	63.7 ± 3.1	63.3 ± 4.0	68.8 ± 4.8	80.4 ± 2.2	54.1 ± 3.0	62.0 ± 3.5	80.5 ± 1.7	76.8 ± 3.0

*Shading is used to indicate significant group differences within each study.*

#### Prenatal Protein Malnutrition

The average number of days to achieve criterion performance in the SAT was compared using an independent samples *t*-test to determine if there was any difference in the rate of acquisition based on prenatal nutritional treatment.

## Results

### Histological Analyses

#### Prenatal Protein Malnutrition

**FIGURE 1:**
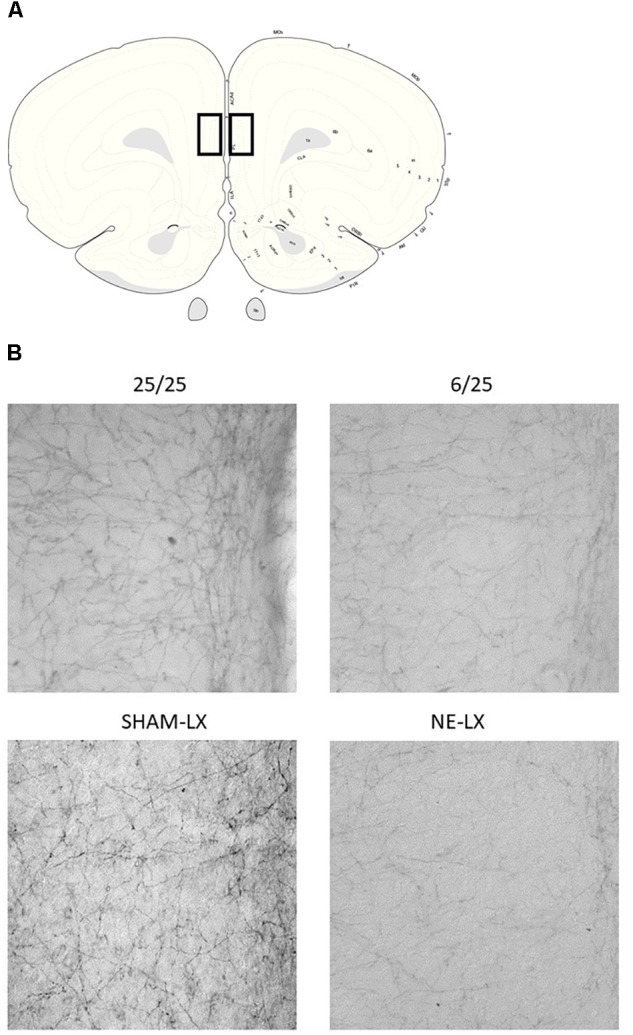
**(A)** A schematic diagram of a coronal slice from the rat brain approximately 3.7 mm anterior to Bregma. Black squares indicate the location of the prelimbic cortex (PL) (Paxinos and Watson, 2007). **(B)** Photomicrographs of the PL showing noradrenergic axons stained for dopamine beta hydroxylase (DBH). The top left image is taken from well-nourished control rats (25/25) and is compared to subjects exposed to prenatal malnutrition (6/25) shown on the right. Subjects from the second study are shown in the row below with SHAM-LX rats shown on the left and NE-LX shown on the right.

**FIGURE 2:**
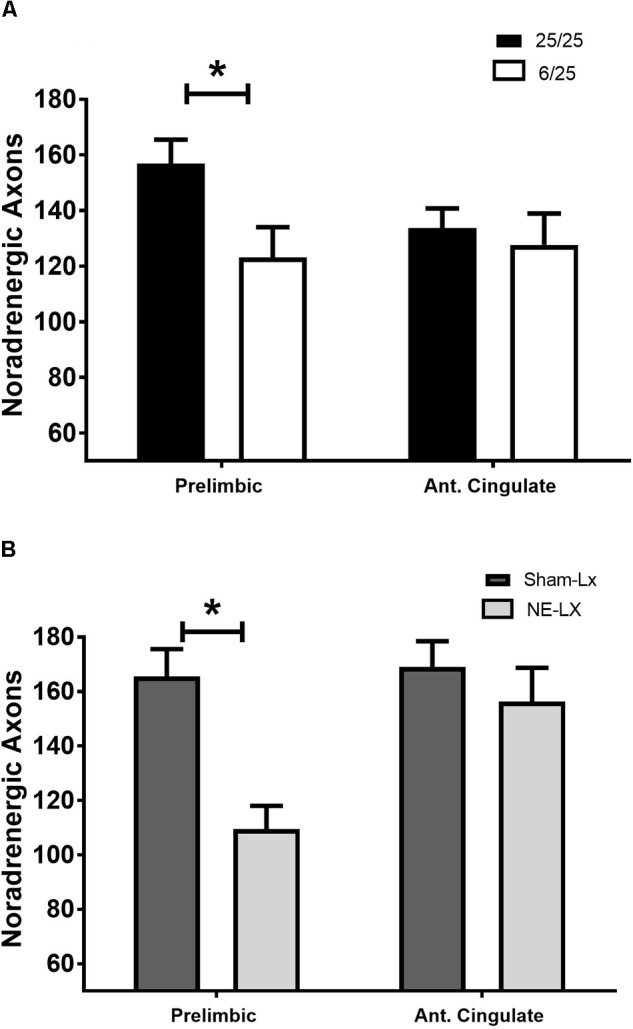
**(A)** Rats exposed to prenatal malnutrition (6/25; white bars) had significantly fewer noradrenergic axons in PL than well-nourished control subjects (25/25; black bars). There was no difference between the number of noradrenergic axons in the ACC between control and malnourished rats. **(B)** Data from experiment two is shown with control subjects indicated by dark gray bars (SHAM-LX) and lesioned rats indicated by light gray bars (NE-LX). Infusions of DBH-saporin produces noradrenergic deafferentation in the PL cortex while sparing fibers in the nearby ACC. Though the extent of noradrenergic damage produced by lesioning the PL was greater than the extent of damage found to result from malnutrition (38% reduction in axons due to lesioning vs. 21.7% after malnutritions), damage following both treatments was limited to the PL. ^∗^ indicates *p* < 0.05.

Prenatal protein malnutrition resulted in significantly fewer DBH positive axons in the PL relative to control values [*F*(1,24) = 5.61, *p* = 0.03; Figure 1A,B, 2A] but did not alter axon density in ACC [*F*(1,24) = 0.26, *p* = 0.61; Figure 2A]. When compared to 25/25 control subjects, the prenatally malnourished 6/25 rats had 21.7 ± 7.0 % fewer DBH positive axons in the PL. These findings did not differ along the rostro-caudal axis in the PL and the ACC showed no significant differences between groups (all *p* > 0.12). There was no difference in cholinergic fiber density as a result of prenatal malnutrition in either PL (25/25: 355.7 ± 30.0; 6/25: 352.0 ± 38.9) or ACC (25/25: 354.0 ± 20.0; 6/25: 370.0 ± 28.0; all *p* > 0.65; data not shown).

#### Noradrenergic Lesions of PL

Damage that resulted from infusion of DBH-saporin in the medial prefrontal cortex produced noradrenergic deafferentation in the PL [*F*(1,20) = 18.13, *p* < 0.001; Figure 1A,B, 2B] but not in the anterior cingulate or orbitofrontal cortices (all *p* > 0.45; Figure 2B). There was no difference in the extent of damage along the rostro-caudal axis or between hemispheres (all *p* > 0.05). On average, the immunotoxin produced a 38.8% ± 2.3% loss of DBH positive fibers at all rostro-caudal levels assessed in PL cortex.

### Prenatal Protein Malnutrition: Effects of Prenatal Protein on Body Weights

Weights prior to the onset of behavioral testing were compared between the two nutrition groups. No differences were found in weight based on the prenatal nutrition group [*t*(25) = 1.37, *p* = 0.19; 6/25: 415.6 ± 11.8 g; 25/25: 438.9.5 ± 11.1 g]. Our aim was to allow subjects to maintain a body weight ≥90% of pre-restriction during behavioral testing. We calculated the lowest post-restriction body weight/pre-restriction body weight for each rat to determine how well they maintained body weight after dietary restriction. After the implementation of food restriction, all rats maintained body weights nearly identical to their pre-restricted weights (6/25 = 100% ± 2.9%; 25/25 = 102% ± 2.0%), and there was no difference between the nutrition groups [*t*(25) = 0.59, *p* = 0.55]. Therefore, weight was not included in further analyses of behavioral performance.

### Baseline SAT

#### Prenatal Protein Malnutrition

**FIGURE 3:**
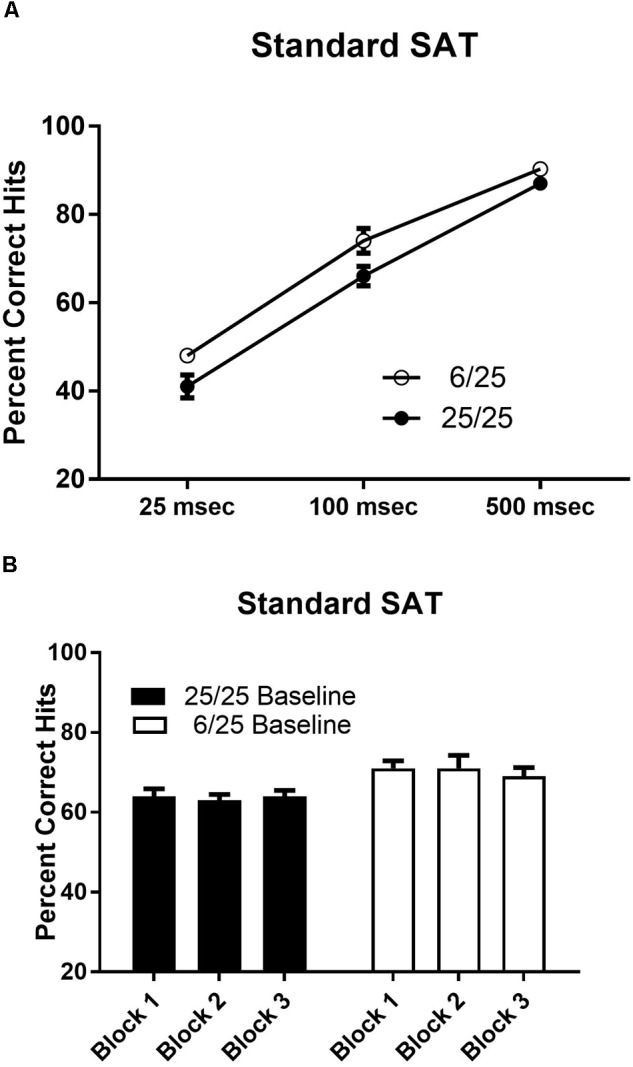
Performance on the baseline SAT task in prenatally well-nourished and malnourished rats. **(A)** The ordinate shows the percent correct responding to signal trials. The abscissa depicts performance at each stimulus length (25, 100, and 500 ms). When accuracy was averaged across all stimulus lengths, 6/25 (empty circles) rats showed statistically significant higher accuracy on signal trials than controls (25/25; filled circles). Means and SEMs averaged across signal lengths are provided in Table 2. **(B)** Blocks of 54 trials are shown on the *x*-axis. Each block contained 27 signal trials and 27 non-signal trials presented in a pseudorandom order so that each target duration was presented on nine trials. There was no change in performance over the course of the testing session in the SAT for either 6/25 (white bars) or 25/25 rats (black bars).

Prenatal malnutrition did not impair acquisition of performance in the standard version of the SAT. The number of days required to achieve criterion performance in the SAT did not differ as a result of prenatal nutritional treatment [*t*(25) = 1.69, *p* = 0.10; range 14–40 days; mean ± SEM; 25.15 ± 1.39]. Rats from the 6/25 group were better at detecting signals than 25/25 rats [*F*(1,25) = 4.78, *p* = 0.04]. Regardless of prenatal nutritional treatment, subjects showed signal length dependent performance on hits [*F*(2,50) = 319.96, *p* < 0.0001; Figure 3A] that was consistent across blocks of testing trials (*p* > 0.7; Figure 3B).

#### Noradrenergic Lesions of PL

One SHAM-LX animal failed to complete post-surgical training so data from that subject were excluded from statistical analyses. For both groups, *n* = 11. Accuracy on signal trials varied by signal duration [*F*(3,60) = 371.95, *p* < 0.0001; data not shown], in both sham- and NE-lesioned rats (*p* > 0.2). Signal detection was unchanged over the course of the testing session and did not differ after NE lesions (all *p* > 0.5). Hits were similar for all days prior to a test of varying attentional demand in both groups (all *p* > 0.17). Noradrenergic lesions did not change the ability of rats to correctly reject non-signals (all *p* > 0.44). There were no other significant main effects of treatment or interactions in the analyses of hits and correct rejections. Both SHAM-LX and NE-LX had similar side biases that were approximately neutral (mean ± SEM; SHAM-LX: 0.43 ± 0.01; NE-LX: 0.44 ± 0.01).

### Task Irrelevant Lights

#### Predictable Distractor

##### Prenatal protein malnutrition

**FIGURE 4:**
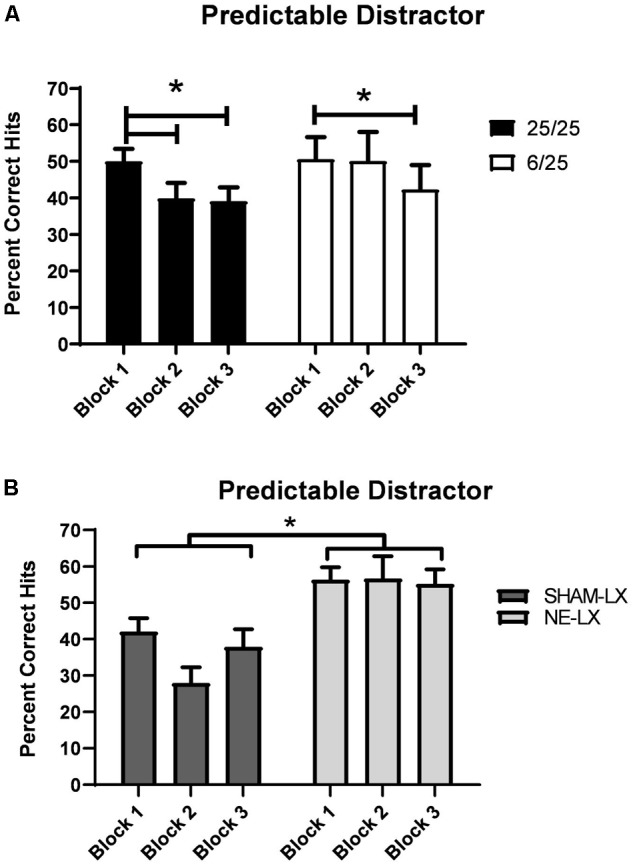
**(A)** 6/25 rats (white bars) were more resistant to cognitive fatigue produced by the presence of a 0.5 Hz visual distractor than 25/25 rats (black bars). Prenatally protein malnourished rats did not show a significant decline in performance under these conditions until the last block of trials. In contrast, 25/25 rats were significantly impaired in detecting signals by Block 2 and their signal detection remained impaired throughout the rest of the testing session. **(B)** Rats with selective noradrenergic lesions of the PL cortex (light gray bars) were more resistant to the effects of a 0.5 Hz visual distractor than controls (dark gray bars) as shown by the higher number of hits (ordinate) over the course of the testing session. ^∗^ indicates *p* < 0.05.

Though the presence of the 0.5 Hz houselight impaired performance regardless of treatment [*F*(1,25) = 59.51, *p* < 0.0001] prenatally malnourished rats were less susceptible to the detrimental effects of the predictable distractor (Table 2). Specifically, well-nourished rats showed more rapid cognitive fatigue in the face of this distractor than did the prenatally malnourished rats [*F*(2,50) = 5.61, *p* < 0.006, see Figure 4A; black bars]. Planned comparisons revealed that 25/25 rats showed significant decreases in performance in Block 2 relative to Block 1 of the distractor session [*t*(16) = 3.14; *p* = 0.006]. This impairment was also observed during the third and final block of testing [Block 1 vs. 3: *t*(16) = 3.88, *p* = 0.001; Block 2 vs. 3: *t*(16) = 0.24, *p* = 0.81]. In contrast to these findings, prenatally protein malnourished rats performed at similar levels of accuracy in Blocks 1 and 2 (*p* = 0.82; Figure 4A; white bars). However, they were significantly impaired by Block 3 relative to the first block of the distractor session [*t*(9) = 4.05, *p* = 0.003] with a trend for poorer performance in Block 3 versus Block 2 [*t*(9) = 2.18; *p* = 0.06]. There were no other significant main effects or interactions found in the hits analyses.

Rats from both nutritional groups emitted more false alarms and fewer correct rejections in the dSAT than the SAT [*F*(1,25) = 8.66, *p* = 0.007; data not shown]. This impairment was largest in the first block of the 0.5 Hz session relative to the baseline session for all rats [Day × Block: *F*(2,50) = 9.25, *p* < 0.001, 𝜀 = 0.86; Baseline vs. 0.5 Hz Block 1: *t*(26) = 5.41, *p* < 0.001]. The rats’ performance did not differ in subsequent blocks (all *p* > 0.05). Prenatal malnutrition did not significantly alter non-signal accuracy or interact with the effects of the distractor (Table 2; all *p* > 0.05).

##### Noradrenergic lesions of PL

Though the presence of the 0.5 Hz houselight (dSAT) impaired performance in both groups [*F*(1,20) = 102.96, *p* < 0.001], noradrenergically lesioned rats were better able to detect signals during this session than SHAM-LX rats [Lesion: *F*(1,20) = 15.52, *p* < 0.001; Lesion × Day: *F*(1,20) = 5.71, *p* = 0.03; Table 2 and Figure 4B]. All subjects maintained signal length dependent performance in both the baseline and dSAT session [*F*(2,40) = 147.86, *p* < 0.001]. There were no other significant main effects or interactions found in the hits analyses.

Rats from both groups emitted more false alarms and fewer correct rejections [*F*(1,20) = 33.07, *p* < 0.001] during the dSAT than the SAT. This impairment was largest in the first block of the 0.5 Hz session relative to the baseline session for all rats [Day × Block: *F*(2,40) = 12.27, *p* < 0.001, 𝜀 = 0.91; SAT vs. dSAT Block 1: *t*(21) = 7.84, *p* = 0.001]. Noradrenergically lesioned rats emitted more false alarms and fewer correct rejections than SHAM-LX rats [Lesion: *F*(1,20) = 5.91, *p* = 0.03, 𝜀 = 0.84]. The presence of the flashing houselight exacerbated this difference [*F*(1,20) = 4.67, *p* = 0.04, 𝜀 = 0.91; Table 2]. The rats tended to respond more on the non-signal lever during the flashing houselight session [Day: *F*(1,20) = 28.47, *p* < 0.001], which was more pronounced in SHAM-LX versus NE-LX rats during the 0.5 Hz distractor (Baseline SHAM-LX: 0.42 ± 0.01; NE-LX: 0.46 ± 0.01; 0.5 Hz: SHAM-LX: 0.26 ± 0.03; NE-LX: 0.40 ± 0.03). This increased non-signal lever responding was confirmed by *t*-tests comparing side bias during the SAT and the dSAT session for both groups [SHAM-LX: *t*(10) = 5.37, *p* = 0.001; NE-LX: *t*(10) = 2.03, *p* = 0.07].

#### Unpredictable Distractor

##### Prenatal protein malnutrition

**FIGURE 5:**
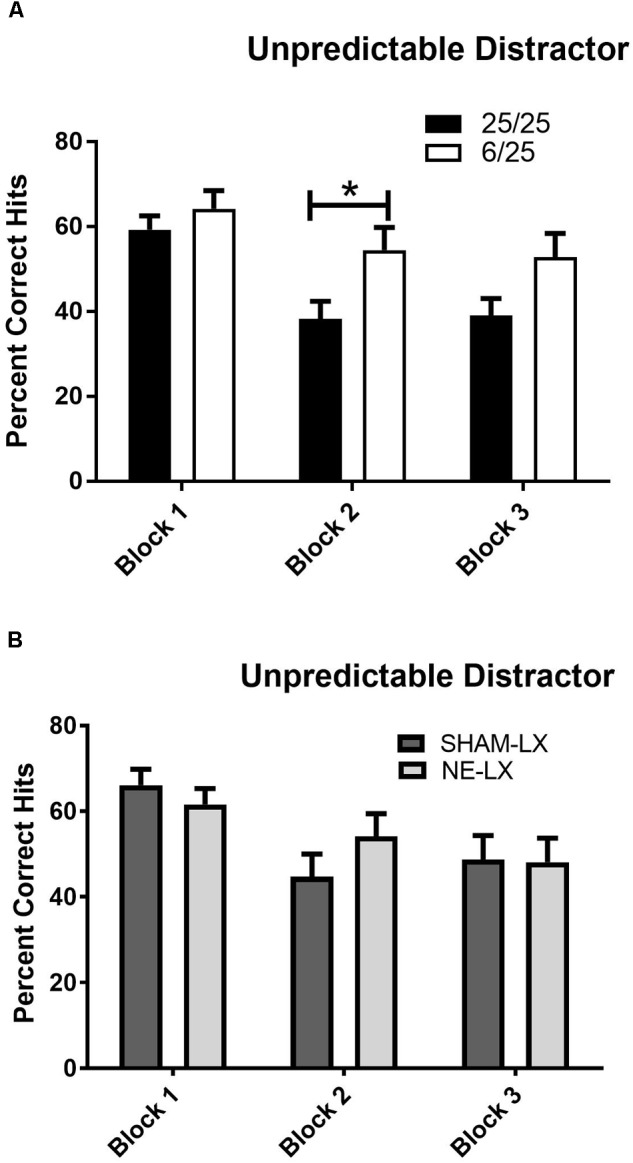
**(A)** 25/25 rats (black bars) were more impaired than 6/25 (white bars) at discriminating visual target stimuli from the unpredictable distractor (0.25, 0.5, 1.0, 1.5, 2.0, or 3.0 s on/off) during the second block of testing. While 6/25 rats were better able than 25/25 rats to maintain signal detection in the presence of this distractor, both groups of rats were impaired when performance in this session was compared to performance in the prior day’s SAT performance as reported in the results. For comparison, see data in Figure 3B. **(B)** Performance in the uSAT was unchanged by noradrenergic lesions with no differences found in the % hits of sham-lesioned (dark gray bars) and noradrenergically lesioned rats (light gray bars). ^∗^indicates *p* < 0.05.

The temporally unpredictable distractor (uSAT) impaired performance of all subjects regardless of prenatal nutritional treatment [Day: *F*(1,25) = 33.42, *p* < 0.001]. Subjects were less impaired in the first block of testing during the uSAT than during subsequent blocks [Day × Block: *F*(2,50) = 7.17, *p* < 0.002; Block 2: *t*(26) = 6.78; Block 3: *t*(26) = 4.77, both *p* < 0.001]. This effect did not vary based on signal duration [Day × Signal × Block: *F*(4,100) = 0.44, *p* = 0.78]. Although there were no significant main effects of prenatal diet (Nutrition) or time on task (Block), a significant interaction was found [Block × Nutrition: *F*(2,50) = 3.71, *p* = 0.03; Figure 5A]. When the unpredictable distractor was first introduced, 25/25 rats and 6/25 rats showed similar performance (Block 1 6/25 vs. 25/25; *p* = 0.38), but 6/25 rats (white bars) were more resistant to the detrimental effects of this distractor over the course of the testing session than 25/25 rats (black bars) in Block 2 of the session [*t*(25) = 2.41, *p* = 0.02], with a similar trend in Block 3 [*t*(25) = 1.93, *p* = 0.06; Figure 5A].

All subjects emitted more false alarms and fewer correct rejections during the uSAT than SAT [Day: *F*(1,25) = 45.91; *p* < 0.001]. All subjects showed an improvement in non-signal performance in later blocks of the uSAT relative to the first block [Block: *F*(2,5) = 27.36; *p* < 00.1; Block 1 vs. Block 2: *t*(26) = 7.37; Block 1 vs. 3: *t*(26) = 7.43; both *p* < 0.001; Block 1: 56.5 ± 2.9; Block 2: 76.3 ± 1.2 Block 3: 78.1 ± 1.5]. There was no difference in correct rejection accuracy based on prior nutritional treatment (all *p* > 0.32; Table 2).

##### Noradrenergic lesion study

Both sham and NE-lesioned rats emitted fewer hits during the uSAT than the SAT [*F*(1,20) = 70.61, *p* < 0.001]. All subjects were less impaired in the first block of testing during the uSAT than during subsequent blocks [Day × Block: *F*(2,40) = 12.79, *p* < 0.001; Block 1 vs. 2: *t*(21) = 4.17; Block 1 vs. 3: *t*(21) = 4.61; both *p* < 0.001; Block 2 vs. 3 *t*(21) = 0.32, *p* = 0.75; Figure 5B]. There was no effect of noradrenergic lesions on uSAT performance (all main effects and interactions, *p* > 0.12; Table 2).

All subjects also emitted more false alarms and fewer correct rejections during the uSAT than the SAT [NE lesion study: *F*(1,20) = 109.48; *p* < 0.001]. All subjects showed an improvement in non-signal performance in the last block of the uSAT [Block 1 vs. 3: *t*(21) = 8.42, *p* < 0.001; Block 2 vs. 3: *t*(21) = 2.58, *p* = 0.017; data not shown]. Noradrenergic lesions did not impair non-signal performance (all main effects and interactions *p* > 0.67; Table 2). No other effects or significant interactions between treatments and any other factor were found in the analyses of signal, non-signal accuracy or side bias.

#### Overlapping Distractor

##### Prenatal protein malnutrition

Distracting stimuli with durations equivalent to target stimuli (oSAT) were only tested in the prenatal malnutrition study. Signal detection of all rats was impaired in the oSAT relative to baseline performance [Day: *F*(1,25) = 61.00, *p* = 0.001]. These effects did not differ between the two prenatal nutritional groups (all *p* > 0.08; Table 2).

**FIGURE 6:**
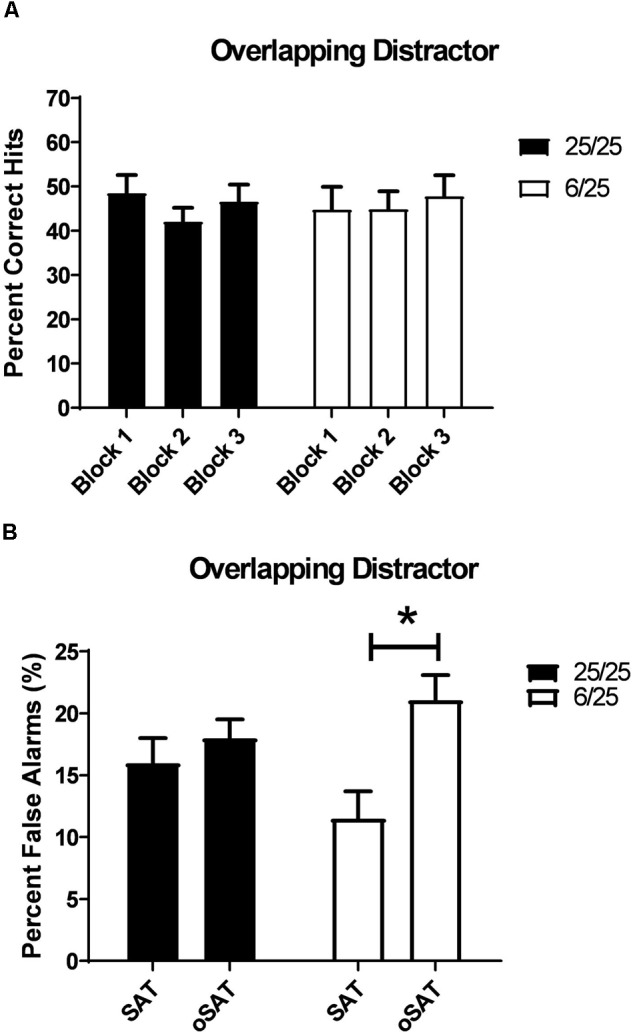
**(A)** The effects of a distracting, visual stimulus that overlapped in duration with targets (500, 100, or 25 ms on/off; oSAT) is shown. All rats were impaired in signal detection during this session when performance was compared to the baseline. For comparison, see data in Figure 3B. This distractor produced similar deficits in all rats regardless of prenatal protein levels. **(B)** Prenatally malnourished rats (6/25, white bars) emitted a greater number of false alarms when the houselight was flashed at durations equivalent to that of target stimuli (oSAT) than when the houselight was constantly illuminated (SAT). Rats from well-nourished mothers (black bars) did not show significantly higher numbers of false alarms during the overlapping distractor session (oSAT) than during than baseline SAT with the houselight constantly illuminated (SAT). These data support the hypothesis that malnutrition contributes to impairments in response inhibition. ^∗^indicates *p* < 0.05.

All subjects were less able to correctly reject non-signal stimuli in the presence of the overlapping distractor [Day: *F*(1,25) = 12.54, *p* = 0.002] despite shifting responding toward the non-signal lever during this session [*F*(1,25) = 19.81, *p* < 0.001; Side Bias oSAT: 0.33 ± 0.014]. In contrast to the effects of the other distracting stimuli, the presence of the overlapping light decreased accurate, non-signal responding in 6/25 rats but not in 25/25 rats (Table 2). When performance in the oSAT was compared to the SAT, rats with prenatal protein malnutrition (6/25; white bars) showed a significant increase in false alarms [Day × Nutrition: *F*(1,25) = 5.82, *p* = 0.02; *t*(9) = 4.47, *p* = 0.002; Figure 6], while well-nourished rats did not [25/25; *t*(16) = 0.86, *p* = 0.40; Figure 6]. There was no difference based on prenatal nutrition in side bias during the overlapping session [*F*(1,25) = 0.08, *p* = 0.78].

#### SAT Without Reinforcement (SATwr)

##### Prenatal protein malnutrition

To test the possibility that nutritional treatment altered subjects’ responses to food reward, we tested SAT performance without food reinforcement. There was no difference based on prenatal malnutrition to effects of withholding reinforcement in the SAT (% hits: all *p* > 0.45; % CR: all *p* > 0.1; Table 2). For all animals, accuracy on both signal [*F*(1,25) = 4.50, *p* = 0.04] and non-signal trials [*F*(1,25) = 15.32, *p* = 0.001] was decreased in this session. The largest drop in accuracy during the SATwr was in response to the 500 and 100 ms signal [Day × Signal Length: *F*(2,50) = 11.84, *p* = 0.0001, 𝜀 = 0.91; Baseline vs. SATwr: 500 ms: *t*(26) = 5.27, *p* = 0.001; 100 ms: *t*(26) = 2.21, *p* = 0.04; data not shown].

The effects of increased time on task exacerbated the effects of withholding reinforcement on signal detection [*F*(2,50) = 8.06, *p* = 0.001, 𝜀 = 0.81]. Though there was no difference in signal detection when the first block of the test session was compared to the same block in the baseline session, performance did differ between sessions in the third block [*F*(2,50) = 8.06, *p* = 0.001, 𝜀 = 0.81; Baseline vs. SATwr: Block 3: *t*(26) = 3.28, *p* = 0.003; data not shown]. The impairments in accuracy were not a result of rats adopting a side bias as all subjects regardless of dietary treatment maintained a neutral side bias during this session. Additionally, while omissions during signal and non-signal trials were significantly higher during the session without reinforcement, they remained extremely low [Signal Trials: *F*(1,25) = 18.89, *p* < 0.001; Baseline: 0.1 ± 0.09; SATwr: 1.16 ± 0.23; Non-signal Trials: *F*(1,25) = 19.73; *p* < 0.001; Baseline: 0.80 ± 0.25; SATwr: 4.46 ± 0.81]. There was no difference in omission rate on signal trials based on prenatal nutrition group [*F*(1,25) = 0.099, *p* = 0.76].

##### Noradrenergic lesion study

There was no difference between NE-LX and SHAM-LX rats during the SATwr (% hits: all *p* > 0.36; % CR: all *p* > 0.13; Table 2), Accuracy on signal trials [*F*(1,20) = 22.19, *p* < 0.001] was decreased during the SATwr relative to SAT for both lesioned and sham-lesioned subjects. The largest drop in accuracy during this session was in response to the 500 and 100 ms signal [Day × Signal Length: *F*(2,40) = 8.05, *p* < 0.001; SAT vs. SATwr: 500 ms: *t*(21) = 7.14, *p* < 0.001; 100 ms: *t*(21) = 5.82, *p* < 0.001; 25 ms: *t*(21) = 1.57, *p* = 0.13]. Though there was no difference in signal detection when the first block of the SATwr session was compared to the same block in the baseline session, performance did differ between sessions in Block 2 and 3 [Day × Block: *F*(2,40) = 14.01, *p* < 0.001; 𝜀 = 0.76; SAT vs. SATwr: Block 1: *t*(21) = 0.62, *p* = 0.54; Block 2: *t*(21) = 4.88, *p* < 0.001; Block 3: *t*(21) = 4.50, *p* < 0.001]. The impairments in accuracy were not a result of rats adopting a side bias as all subjects maintained a neutral side bias during this session (SATwr = 0.44 ± 0.02). Additionally, while omissions during signal trials were significantly higher during the session without reinforcement, they remained extremely low [*F*(1,20) = 92.27, *p* < 0.001; Baseline: 0.13; No reinforcement: 1.74 ± 0.18]. Noradrenergic lesions did not alter the response to withholding of reinforcement on hits or omissions (all main effects and interactions *p* > 0.15; Table 2).

Withholding reinforcement decreased non-signal accuracy [*F*(1,20) = 43.71, *p* < 0.001; Table 2] in all subjects. Non-signal performance declined significantly over the course of the SATwr session [*F*(2,40) = 4.28, *p* = 0.02] with a large decrease in accuracy when Block 2 was compared with Block 1 [*t*(21) = 3.25, *p* = 0.004] but no further decline in performance in Block 3 relative to Block 2 [*t*(21) = 0.98, *p* = 0.34]. Omissions on non-signal trials were higher when reinforcement was withheld but remained low [Day: *F*(1,20) = 102.07, *p* < 0.001; Baseline: 0.54 ± 0.13; No reinforcement: 6.62 ± 0.62]. There were no other main effects or interactions in the analyses of the effects of noradrenergic lesions on performance in the SATwr.

## Discussion

The present study is the first, to our knowledge, to directly compare the effects of prenatal protein malnutrition to selective noradrenergic deafferentation of the PL cortex. Few studies have assessed the effects of noradrenergic damage on sustained attention in the rat, and published results have been inconclusive. Prior work by McGaughy et al. (1997) showed that lesions to the dorsal noradrenergic bundle failed to impair performance on the SAT or dSAT. Assessments of lesions in this study were based on homogenates of the entire frontal cortex and failed to differentiate damage within prefrontal sub-regions so the extent of damage to PL is unknown. It is possible the failure to dissociate the attentional performance of lesioned and sham-lesioned rats results from the relative sparing of PL. Previous studies by Carli et al. (1983) showed that lesions to the dorsal noradrenergic bundle sufficient to deplete >80% of cortical norepinephrine did not impair baseline performance in a visual search task. These subjects were, however, impaired by the interpolation of a loud, white noise distractor (Carli et al., 1983) which was interpreted as an increased sensitivity to the stress-related effects of this distractor. The present study did not directly examine the effects of distraction in conjunction with stress, but prior studies have shown that prenatal malnutrition confers a vulnerability to stress concomitant to alterations in cortical monoamines (Mokler et al., 2007). Future studies will be aimed at determining how stress interacts with distractibility in both noradrenergic lesioned and malnourished subjects.

Prenatal malnutrition and noradrenergic deafferentation of the PL did not affect performance significantly in the baseline SAT. This is consistent with prior work in lesioned rats (Carli et al., 1983; McGaughy et al., 1997). Additionally, neither group of experimental animals was found to be more sensitive to withholding reinforcement. This preservation of function confirms prior reports that malnutrition produces specific attentional impairments rather than global difficulties in understanding response rules, bottom-up problems in perception or cognition secondary to primary dysfunction in sensitivity to reinforcement contingencies (Morgane et al., 1993; Tonkiss et al., 1993; McGaughy et al., 2014).

Prenatally malnourished rats were less susceptible to the effects of a predictable and unpredictable visual distractor than well-nourished control subjects. Prenatally malnourished rats maintained higher target detection rates for a greater portion of the testing session than control subjects in tests of distractibility. These findings may seem counterintuitive as they suggest that prenatally malnourished rats perform better in the face of distraction. However, these data, in conjunction with previous data (Tonkiss et al., 1993; Strupp and Levitsky, 1995), support the hypothesis that prenatal malnutrition produces cognitive rigidity and this confers resistance to distraction. While behavioral inflexibility may be beneficial when disregarding a distractor, it was found to be detrimental when a subject was required to change its strategy and to attend a novel stimulus dimension in a test of attentional set shifting (McGaughy et al., 2014), demonstrating that normal attention requires adaptation to current cognitive demands.

The results of the NE lesion study show that acute noradrenergic deafferentation of the PL cortex produces similar, but distinctive, results to prenatal protein malnutrition. Noradrenergic lesioned rats were more resistant to the effects of the predictable distractor than controls, but the groups did not differ in their responses to the unpredictable distractor. The present study also shows that prenatal protein malnutrition results in fewer noradrenergic afferents to PL cortex but does not impact afferents to the ACC or cholinergic afferents to any prefrontal sub-region sampled. These findings are in line with recent data showing noradrenergic innervation of the cortex is topographically discrete with less overlap in cortical innervation than previously assumed (Chandler et al., 2013). It is perhaps unsurprising that adult lesions restricted to PL cortex failed to fully replicate the neurodevelopmental insult. Though the extent of noradrenergic damage produced by lesioning the PL cortex was greater than the extent of damage found to result from malnutrition (38% vs. 21.7% reduction in fibers), prenatally malnourished rats were resistant to both predictable and unpredictable distractors, while lesioned rats were resistant only to the predictable distractor suggesting malnutrition produces a more severe cognitive rigidity than acute noradrenergic lesioning. However, a closer look at these data, show remarkable similarities between the performance of prenatally malnourished and lesioned rats in the presence of the unpredictable distractor (uSAT 6/25: 57.1 ± 4.1; NE-LX: 54.6 ± 3.2). *Post hoc* comparison of the well-nourished and sham-lesioned rats failed to reveal a statistically significant difference in the performance of these groups during the distractor (uSAT: 25/25: 45.5 ± 3.0; SHAM-LX: 53.2 ± 4.9), but the sham-operated controls in Experiment 2 had more variable performance in the presence of the unpredictable distractor. It is therefore possible that the differences in interpretation of the data may be due to differences between control groups rather than a critical difference in the effect of prenatal malnutrition and noradrenergically lesioned rats.

Previous research has documented increased levels of brain catecholamines after perinatal food restriction and protein malnutrition (Burns and Brown, 1977; Ramanamurthy, 1977; Molendi-Coste et al., 2006). However, adults that had previously been fostered to well-nourished dams *did not* show increased cortical norepinephrine (Soto-Moyano et al., 1999). Additionally, when found these higher than normal levels of cortical norepinephrine have been shown to decrease by adulthood (Stern et al., 1975; Chen et al., 1997; Soto-Moyano et al., 1999). Previous research has also shown that these changes were regionally specific (Stern et al., 1975; Chen et al., 1997). An *in vivo* microdialysis study of the ventral mPFC, including the PL, has revealed decreased levels of both norepinephrine and dopamine in the right hemisphere of the PL (Mokler et al., 2019). At present, we are not able to reconcile the unilateral extent of decreased cortical efflux with decreased noradrenergic axons occurring bilaterally. The changes in cortical norepinephrine do not seem to result from changes in the locus coeruleus which is unchanged by prenatal protein malnutrition in male rats (King et al., 1999). However, it is important to note that the locus coeruleus is sexually dimorphic (Pinos et al., 2001; Bangasser et al., 2016). Because data from human studies of early life malnutrition have found the attentional and emotional impairments (Galler and Ramsey, 1989; Galler et al., 1990, 2012; Waber et al., 2014) are equally prevalent in males and females, we have focused our pre-clinical studies on male rats. However, the effects of prenatal malnutrition on female subjects in this animal model remains an underexplored and critical question. From these studies and others, it is apparent that prenatal protein malnutrition alters the development of the noradrenergic systems centrally and peripherally, and these effects can vary depending on the severity of malnutrition, the timing of the insult, the age of the animal at testing, and the brain region studied. Though the precise mechanism of these changes remains unresolved, prenatal malnutrition has been shown to produce epigenetic changes in both humans and a rodent model (Peter et al., 2016). Specifically malnutrition lowers transcription of the catechol-*O*-methyltransferase gene in the humans and the prefrontal cortex of male rats which may contribute to altered catecholamine signaling in the cortex of malnourished subjects (Peter et al., 2016).

To our knowledge, the present experiment is the first-time noradrenergic fiber density has been studied in the prefrontal cortex after prenatal malnutrition, and, as these regions are more topographically restricted than previously assumed (Chandler and Waterhouse, 2012), it may not be unexpected that the fiber density varies across prefrontal subdivisions. The assessment of axons in the present study found that the density of axons is similar across prefrontal sub-regions. This is similar to prior studies of the number of axons (Cerpa et al., 2019). In contrast, the density of noradrenergic varicosities has been shown to be higher in the ACC than the more ventral regions of the medial prefrontal cortex, i.e., PL combined with IL (Agster et al., 2013). Interestingly the study by Cerpa et al. (2019) founds a substantial overlap between measurements of axon density and varicosities. This suggests the difference is not related to the dependent measures but additional studies where both axons and varicosities are assessed are needed to resolve this point. Another source of the discrepancy between our study and Agster’s (Agster et al., 2013) may be due to a nearly 2 mm difference in sampling of ACC between the studies. The current experiment and other work has focused on pre-genu portions of the ACC in the rat based on functional homologies of this region to the dorsal ACC in humans (Milham and Banich, 2005; Orr and Weissman, 2009; Newman and McGaughy, 2011; Newman et al., 2015), but future studies will be aimed at assessing caudal regions of the ACC as well to determine how malnutrition impacts them.

In contrast to the resistance to distraction found with other distractors, the overlapping distractor, revealed a unique susceptibility of the prenatally malnourished rats. Malnourished rats were more likely than control subjects to emit false alarms during the session with the overlapping distractor while target detection did not differ between the groups. The increase in false alarms may reflect additional problems in response inhibition caused by the prenatal insult that are revealed only under conditions of high perceptual overlap. These data are in line with previous research by this group showing mild, but consistent, impairments in response inhibition resulting from prenatal protein restriction (Tonkiss et al., 1993; McGaughy et al., 2014). The difference between the malnourished subjects’ response to the overlapping distractor and other distracting stimuli is likely to reflect the impact of prenatal protein malnutrition on cortical regions beyond the PL. Though the integrity of the prefrontal cortex is required to disregard distracting stimuli (Newman et al., 2008; Berry et al., 2014), the ability to differentiate perceptually identical distractors from targets relies on the parietal cortex (Buschman and Miller, 2007). Though assessments of parietal cortex were not undertaken in the present study, future work will address the impact of prenatal protein malnutrition on this region critical to attentional control. Unfortunately, this variant was not tested in the assessment of PL lesioned rats, so direct comparisons on this particular measure cannot be made between the two studies.

Neither prenatally malnourished nor noradrenergically lesioned rats were impaired in the baseline version of the task. These data are consistent with findings in the prenatally malnourished rats that cholinergic afferents to prefrontal cortex were unaffected by malnutrition. Phasic activity of the cholinergic system in the prefrontal cortex is critical to allowing subjects to shift between internally and externally driven attention (Howe et al., 2013), while tonic levels are hypothesized to allow target detection (Sarter and Paolone, 2011). Though our histochemical analyses provide only a static measure of the integrity of the prefrontal cholinergic system, the preservation of baseline performance on the SAT is consistent with our finding of unaltered cholinergic afferents.

The current data show that rats with fewer noradrenergic axons in PL resulting from lesioning or prenatal malnutrition are less distractible than controls. Early-life malnutrition produces cognitive rigidity in our rodent model in the present study and in prior work (McGaughy et al., 2014; Tonkiss et al., 1993) that parallel reports of cognitive rigidity and reduced response inhibition in a human population with histories of early childhood malnutrition (Galler et al., 2012; Waber et al., 2014; Peter et al., 2016). Drugs that increase cortical norepinephrine, e.g., selective reuptake inhibitors, have consistently been shown to improve cognitive flexibility and response inhibition in humans (Chamberlain et al., 2007) and in rodent models that assess the effects of noradrenergic lesions (Robinson et al., 2007; Newman et al., 2008; Cain et al., 2011; Harvey et al., 2013). Increased levels of cortical norepinephrine have been hypothesized to be necessary to broaden attention when response strategies are no longer successful (Aston-Jones and Cohen, 2005; Bouret and Sara, 2005; Berridge et al., 2012). Because malnutrition impacts noradrenergic afferents in PL cortex and alters cognitive flexibility as well as response inhibition (McGaughy et al., 2014), future studies should be aimed at assessing the efficacy of drugs and other interventions that augment noradrenergic function in vulnerable populations. The current study is limited by the focus on maternal malnutrition. Because our aim is to translate our findings to human populations where paternity may be difficult to determine, we have focused on understanding how maternal malnutrition impacts attention and cognition. It is clear that nutritional status of the father is also an important question to address in future studies. An additional limitation is the use of only male subjects. Human studies have shown that prenatal malnutrition impacts attentional performance males and females equally (Galler et al., 2012; Waber et al., 2014) but future studies in our animal model will include females to directly address this in the current animal model. In summary, prenatal malnutrition produces selective impairments in attention and response inhibition that are hypothesized to result, at least in part, from the lower levels of noradrenergic afferents in PL cortex. These deficits are not secondary to basic perceptual or learning impairments and reflect the unique vulnerability of the prefrontal cortex to this neurodevelopmental insult.

## Author Contributions

LN contributed to the acquisition and analyses of the data. JB contributed to the acquisition of the data. DM, JG, and JM contributed to the conception, design, analyses, and interpretation of work. AR contributed to the analyses of the data and manuscript preparation.

## Conflict of Interest Statement

The authors declare that the research was conducted in the absence of any commercial or financial relationships that could be construed as a potential conflict of interest.
